# Applying a novel visual-to-touch sensory substitution for studying tactile reference frames

**DOI:** 10.1038/s41598-021-90132-7

**Published:** 2021-05-20

**Authors:** Or Yizhar, Galit Buchs, Benedetta Heimler, Doron Friedman, Amir Amedi

**Affiliations:** 1 Department of Cognitive and Brain Sciences, The Hebrew University of Jerusalem, Jerusalem, Israel; 2 Baruch Ivcher School of Psychology, Interdisciplinary Center Herzliya, Herzliya, Israel; 3 Center of Advanced Technologies in Rehabilitation (CATR), Sheba Medical Center, Ramat Gan, Israel; 4 Sammy Ofer School of Communications, Interdisciplinary Center Herzliya, Herzliya, Israel; 5 The Ruth & Meir Rosenthal Brain Imaging Center, Interdisciplinary Center Herzliya, Herzliya, Israel

**Keywords:** Psychology, Sensorimotor processing, Sensory processing

## Abstract

Perceiving the spatial location and physical dimensions of touched objects is crucial for goal-directed actions. To achieve this, our brain transforms skin-based coordinates into a reference frame by integrating visual and posture information. In the current study, we examine the role of posture in mapping tactile sensations to a visual image. We developed a new visual-to-touch sensory substitution device that transforms images into a sequence of vibrations on the arm. 52 blindfolded participants performed spatial recognition tasks in three different arm postures and had to switch postures between trial blocks. As participants were not told which side of the device is down and which is up, they could choose how to map its vertical axis in their responses. Contrary to previous findings, we show that new proprioceptive inputs can be overridden in mapping tactile sensations. We discuss the results within the context of the spatial task and the various sensory contributions to the process.

## Introduction

How does body posture influence the way we interpret and perceive our surroundings? Our constant physical interaction with the world requires a continuous update of the body’s location in space, its relation to other objects, and its relation to itself (e.g., the relative positions of body parts in motion). These varied representations of the body form a conscious perception of the environment and play an essential role in action planning. Think for instance of driving a car with a steering wheel in your hand—sensory information about the wheel gives rise to a coherent perception of its function and leads to a set of possible actions that one can perform with it. First, we access the wheel’s physical dimensions through tactile stimulations received on our palms that form an anatomical reference frame. To steer the car, we map this information into a different reference frame by integrating spatial visual information that fits the wheel’s functional use (e.g. the sidewalk is to *right*, the opposite lane is to *left*)^1,2^. The mapping results in the adoption of an allocentric reference frame that is independent of the body, relating objects’ dimensions to external anchors, or of an egocentric reference frame, relating objects’ positions to one’s body^3,4^. We can map tactile stimulations into many allocentric or egocentric reference frames with the ultimate selection depending on the actions that precede or follows the sensation^5,6^, the gravitational dimensions of the environment^7–10^, and the general position of the body^2,4,11^.

Which cognitive mechanisms drive the mapping of tactile information into reference frames? One influential view considers the mapping as part of a wider process of acquiring sensorimotor contingencies^2,8,12,13^. According to this theory, perception emerges through the experience of many co-patterns of incoming sensory signals coupled with outgoing motor actions towards the stimulus. In the context of mapping tactile sensations, we learn different reference frames from exposure to tactile stimulations that integrate with visual and proprioceptive cues to execute diverse actions. Thereafter, many reference frames are accessible with different probability weights that change with ongoing sensorimotor experiences, which we then implicitly retrieve in the mapping process^2,3,13^. Supporting studies show that a change to body posture, gaze, or object’s position in space triggers a gradual adaptation period marked by inconsistent reference frame selections as participants integrate new sensory information^4,10,14,15^. Over time, participants’ reference frame choice becomes more robust as new contingencies are established^2,10,16,17^. Yet, the description of mapping tactile sensations to reference frames as a byproduct of sensorimotor contingencies overlooks the distinct contribution of proprioception to the process, which is less studied and harder to isolate. In particular, previous studies include complex spatial and cognitive tasks such as the need to spatially locate the object after changing postures^2,5,13^, manual delivery of tactile stimuli that bias participants’ responses^4,14,15^, or use of mirrored alphabet letter (e.g., ‘p’ and ‘q’)^4,10,15,18^. Such factors can influence the perception of tactile stimuli and are thus separate from the effects of posture. Furthermore, many experimental paradigms included visual inputs that have a particularly strong influence on reference frame selection^14,17,19–22^.

**Figure 1:**
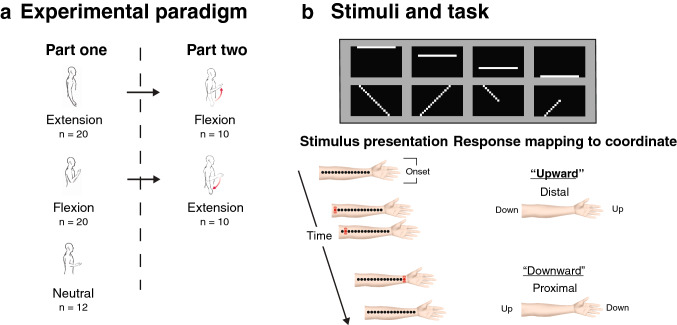
Experimental design and setup. (**a**) Participants (n = 52) were randomly allocated to one of three postures—Extension, Flexion, or Neutral. At the end of part one, 20 participants switched their posture from Flexion to Extension or vice versa. (**b**) Participants had to report the location or orientation of an image that included a diagonal or horizontal line, and which they did not see before. The images are set in opposite pairs, each flipped on the horizontal axis compared to its counterpart. A program temporally scans the 2D images from left to right and transforms each column into an array of evenly-spaced vibrotactile actuators that are on the inner arm. The stimulus presentation shows how an image of a diagonal line translates into a series of vibrations on the inner arm, time represents the x-axis and the physical location of the tactile sensation represents the y-axis (the red dot represents an active vibrator). An image of a horizontal line would result in the activation of a single vibrator, and the length of the line can be inferred from the vibration duration. The vertical orientation of the image is deliberately ambiguous and the line can be perceived as going up or down. We classified responses that matched tactile sensations closer to the wrist to the visual image’s upper bound as distal (“line is going up” in our example), and responses that fit the visual image’s upper bound as closer to the elbow as proximal.

In the current study, we tested the effects of switching body postures on the mapping of tactile sensations to a position in a visual image. To disentangle the contribution of proprioception from other factors, we built a visual-to-tactile Sensory Substitution Device (SSD)^23–25^ that transforms 2D black-and-white images (see Fig. 1b) into a series of tactile vibrations delivered on the inner arm (similar to EyeMusic^26^) of blindfolded participants. A computer program temporally scans the image column by column, from left to right. At each time point, the program translates a column of 15 pixels to an array of 15 evenly-spaced vibrators on the arm. Thus, time represents the horizontal axis (i.e., the first vibrations are from the left part of the image), and the physical location of vibrations represents the vertical axis. We positioned the device on the inner arm of participants such that it moves together with the arm and thus nullifies the need to actively locate the device after changing posture. With this unique setup, we asked blindfolded participants to perform simple spatial tasks by matching the sequence of tactile stimuli to a visual image. Crucially, we asked some participants to change their arm posture between trial blocks and perform the same spatial tasks. The direction of the vertical task was ambiguous, participants were not told which end of the device was up and which was down and were not exposed to the images beforehand. The perception of the visual image vertical axis was then derived from participants’ responses and combined with posture to uncover a choice of reference frame.

According to a sensorimotor contingencies prediction, after switching postures new proprioceptive cues will gradually integrate with a stored body representation that will produce an adaptation and learning period, characterized by less consistent responses. Results in this direction will suggest that the sensorimotor account holds even when vision is absent and with low task demands. In other words, it would show the strong influence of new proprioceptive signals on the mapping of tactile sensations to the visual image. An alternative hypothesis could regard proprioception as a particular and less dominant sensory modality, one which we are less consciously aware of^27^. This would mean that participants adapt fast to new postures, as top-down information overrides incoming bottom-up, potentially conflicting, proprioceptive cues. Such results would diverge from a pure sensorimotor contingency description of mapping tactile sensations, and focus attention on the differential contribution of proprioception^2,4,5,10^.

## Methods

### Participants

A total of 52 healthy participants took part in the experiment (age 32.4 ± 11.8, average and standard deviation; 31 females; 46 right-hand dominants). None of the participants reported any hearing or proprioceptive impairment. *Ethics.* Interdisciplinary Center Herzliya (IDC) institutional ethics committee approved the experiment which falls under their guidelines and regulations, all participants signed an informed consent form and pair for their participation in the study. Participants had no prior familiarity with the device, the algorithm, nor any other SSD.

### The “Tactile Glove”: device description

The “Tactile Glove” is a custom-built Sensory Substitution Device (SSD) that conveys visual information from a 2D image into vibrotactile stimulations. The glove consists of a row of 15 standard coin vibration motors (8 mm diameter) set on the participant’s inner arm, another vibrator on the index finger acts as a precursor. A five Volt logic supplies each actuator via an interface with a data acquisition module (iUSBDAQ-U120816, HYTEK Automation). An accompanying algorithm (written in C#) down-samples 2D images to a 15-by-25-pixel grayscale image, with white pixels denoting objects (e.g. lines or shapes). The binary image is temporally scanned from left to right, column by column using a sweep-line approach. For each white-colored pixel detected in a column, an actuator simultaneously vibrates on the inner arm. This procedure results in the image’s Y-axis represented by the spatial location on the arm, and the X-axis represented by timing (e.g., the participant senses first the left part of the image). Each stimulus comprises a 300 ms precursor cue, a short 100 ms pause, and another 150 ms spent on each column of the image. The Tactile Glove was always positioned on participants’ dominant arm.

### Stimuli and postures

The stimuli consisted of 8 black and white images: four images depicted horizontal lines and another 4 depicted diagonal lines (see Fig. 1b). The images are paired, such that each image has a corresponding flipped pair on its vertical axis. In this manner, the images’ location, length, or orientation would not bias the perception of the up or down of the vertical axis. We used three postures as experimental conditions (Fig. 1a): Arm Extension, with the palm facing outward in full supination; Arm Flexion, with participants’ elbow leaning on an adjacent table, palm facing toward the face; An in-between position, named a Neutral posture, with the arm placed on the table and the palm facing the ceiling. We visually verified throughout the sessions that participants maintain their postures during experimental blocks.

### Procedure

In this study, we investigated the properties of reference frame preferences when relying solely on proprioceptive cues. To this aim, we used a visual-to-tactile SSD that transforms 2D black and white images into a series of vibrations delivered on the inner arm. A program scans the image horizontally from left to right (X-axis), capturing a single column (Y-axis) of pixels at a discrete-time point. Each pixel in the column corresponds to one of the 15 vibrators that make up the device. If the pixel is white, and thus part of an object, the corresponding actuator vibrates. The procedure results in a sequence of temporal vibrotactile stimulations, where time substitutes the X-axis of the image, and the location of vibrations on the inner arm substitutes the Y-axis (Fig. 1b). We instructed blindfolded participants to place their arm in one of three postures—Extension, Flexion, or Neutral (Fig. 1a) but did not provide any instructions on the vertical axis’ direction (which way is up and which is down). In each experimental trial, we presented participants with an image of a line (Fig. 1b) and asked them to report on the line’s spatial location or orientation (towards the upper/lower bound of the picture), focusing on the perceived vertical axis of the stimuli. Note that participants were not shown the visual images beforehand and did not receive any information on their content. We fitted the device on blindfolded participants’ dominant arm and gave a short introduction about the device and experimental process, followed by two introductory pre-test stimuli. Participants had to report the orientation or spatial position of the stimuli. For horizontal line stimuli, the question was *“Is the stimulus located on the upper or lower part of the image?”*, and for the diagonal line stimuli, *“Does the stimulus have a downward or upward slope?”.* The experimenter did not provide any feedback on participants’ responses. Every trial block (i.e., posture) included 16–24 randomized trials. Each trial had three stimulus repetitions with a 200 ms interstimulus interval, followed by a verbal response from the participant. In part one (Fig. 1a), we assigned participants to the Neutral (n = 12), Extension (n = 20), or Flexion posture (n = 20). In part two, we asked 20 participants who performed the Flexion and Extension conditions to switch their posture before completing another block of trials with the same task. To reduce implicit biases, we told participants that switching posture is necessary to cut fatigue. At the end of each experiment (n = 52), we asked participants *“how did you decide what is up and what is down in the image?”*. We deliberately did not ask participants about the relation to the arm or the gravitational axis. 22 Sample responses are in Supplementary Table S1. In some instances, we presented participants with another stimulus right before removing the blindfold and asked them to draw the image on a piece of paper (see Supplementary Fig. S1).

### Statistical analyses

We categorized participants’ responses on their perception of the line’s vertical axis, referred to here as a coordinate selection. We first defined responses based on the anatomical terms of location. We defined distal responses as a perception of the visual image’s upper bounds going away from the trunk and towards the hand. Proximal responses are the perception of the line’s upper bounds located towards the trunk and away from the hand (Fig. 1b). When comparing experimental parts, we classified responses on their implied reference frame mapping, which combines the coordinate selection with the arm’s posture during a given trial block. A gravitational mapping that fits the axis of the room or the position of the trunk and head. An arm-centered mapping that is anchored to the inner arm’s anatomy (e.g., the wrist up and the elbow is down). For the group-level analysis, we averaged participants’ proportion of responses that fit a coordinate or a reference frame (e.g., distal/gravitational). As responses are binary and complementary, we analyzed only the proportions of distal responses for coordinate selection and gravitational responses for reference frame preference. We first conducted a one-sample t-test to determine if the proportion was greater than chance. When computing the differences between groups, we used a non-paired two-sample t-test. As the result can be significantly below or above chance the statistical tests were double-sided, we adjusted the reported p-values accordingly. We performed the group-level analysis on consistency with a t-test and a comparison to chance level. To compare between experimental parts, we used a factorial two-way repeated-measures ANOVA, and to compare between postures in part one we used a one-way ANOVA (for full results see Supplementary Tables S2–4). All data sets passed Bartlett’s test to and confirm with the assumptions on variation. For the subject-level analysis, we used a Normal distribution approximation and then conducted a t-test. Statistical inferences were corrected for multiple comparisons using False Discovery Rate (α = 0.05). To examine the consistency in participants’ responses we measured the absolute difference between the proportion of distal response and compared it to chance level (50%). For group-level analysis, we compared the group’s average consistency to chance level using a t-test. We conducted all the above-mentioned statistical analyses using the MATLAB software (MathWorks). Sample sizes are based on the single-subject analysis, such that the number of overall trials would be sufficient for a normal approximation to the binomial (${\text{n > 9}}\left( {\frac{{{1} - {\text{p}}}}{{\text{p}}}}\right){\text{, p = 1}} - {\text{p = 0}}{.5}$), which allows for a standardized t-test. As the single subject responses follow a parametric distribution that fits a normal distribution so does the group average responses. We did not exclude participants nor samples from the study.

## Results

### Initial posture affects coordinate selection

#### Part one

**Figure 2:**
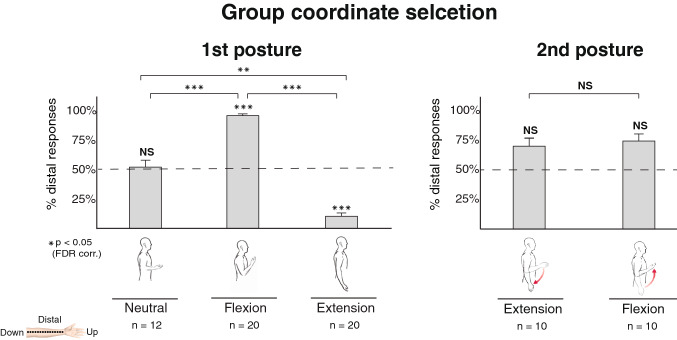
Group results on coordinate selection for parts one and two. We calculated the average proportion of responses fitting a distal mapping to the upper part of the image by posture and compared each one to chance (bars indicate the standard error; dashed line indicates chance level). In part one, the average distal proportion was above chance in the Flexion group, below chance in the Extension condition (i.e., a proximal preference), and insignificant in the Neutral condition. The differences in responses show the implicit effect of posture on coordinate selection. After switching postures in part two, average distal responses in the Flexion and Extension conditions were not different from chance.

Participants in the Neutral posture (Fig. 2) showed no significant distal preference (M = 51.7%, SE = 13.1%, mean group proportion, standard error) compared to chance (t (11) = 0.126, *p* = 0.451, 95% CI [− 0.26, 0.3]). In the Flexion condition (Fig. 2), average responses matching a distal selection), a somatotopic location closer to the wrist, were high (M = 95.3%, SE = 1.8%) and significantly larger than chance (t (19) = 24.13, *p* < 0.001, CI [0.42, 0.49]). In contrast, a low number of responses in the Extension posture (Fig. 2) matched a distal selection (M = 10.5%, SE = 5%) that were significantly smaller than chance (t (19) = − 7.88, *p* < 0.001, CI [− 0.49, − 0.3]). An ANOVA on distal reponses with a between-factor of posture found a significant difference between the three groups (F (2,49) = 48.94, *p* < 0.001, ${{\upeta }^{2}}$ = 0.67). Next, we performed pairwise comparisons among the conditions with a non-paired t-test. Distal responses in the flexion posture were significantly higher than Extension (t (38) = 15.9, *p* < 0.001, CI [0.74, 0.96]) and Neutral (t (30) = 4.02, *p* < 0.001, CI [0.16, 0.67]) postures. Also, responses in the Neutral posture were significantly higher than the Extension posture (t (30) = 3.34, *p* = 0.002, CI [0.21, 0.66]).

### Switching postures modulates reference frame mapping

#### Part two

**Figure 3:**
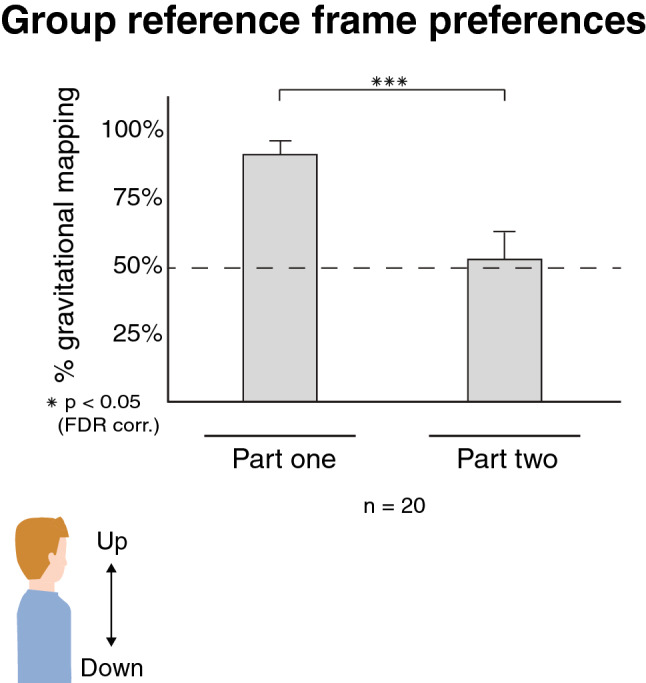
Group analysis on the average proportion of responses matching a gravitational-based tactile to visual mapping (bars indicate the standard error; dashed line indicates chance level). There was a significant difference in the mapping preferences between part one and part two. Thus, switching postures change participants’ mapping of touch to the visual image.

Participants were first assigned either the Flexion or Extension posture in part one and were then instructed to switch to the opposite posture. Following the posture switch, participants’ preferences in coordinate selection diverged from part one (Fig. 2). Responses fitting a distal coordinate did not differ from chance for the Flexion posture (M = 74.4%, SE = 12.3%, t (9) = 1.99, *p* = 0.078, CI [0, 0.48]) nor for the Extension posture (M = 70%, SE = 13.6%, t (9) = 1.48, *p* = 0.174, CI [− 0.07, 0.47]). A non-paired comparison between the postures showed no significant differences (t (18) = 0.23, *p* = 0.814, CI [− 0.34, 0.43]). We next explored the disparity between the results of part one and part two. We considered the differences as an indication of mapping in line with the gravitational axis in part one, and that switching postures before part two affects this assumed mapping. To test this hypothesis, we calculated the proportion of responses matching the gravitational axis. These corresponded to distal responses in the Flexion posture and proximal responses in the Extension posture. We then analyzed the effect of switching postures on gravitational mapping (Fig. 3) with a repeated-measures ANOVA with a within-factor of experimental part and a between-factor of posture order (Extension-Flexion or Flexion–Extension). The test revealed a main effect of part (F (1,18) = 13.97 *p* = 0.001, ${{\upeta }^{2}}$ = 0.35) that shows a significant difference in the responses after switching postures. We also observed some interaction on the factors, which shows the order of postures modulated participants’ behavior following the switch (F (1,18) = 7.65, *p* = 0.013, ${{\upeta }^{2}}$ = 0.19).

### Participants are consistent before and after switching postures

We wanted to test if participants’ responses are consistent within each experimental block, regardless of posture or mapping destination (see methods). To do so, we calculated a consistency estimate of the absolute difference between the proportion of a participant’s distal response and chance level (50%). For the group, a t-test revealed that the confidence level was well above chance for part one (t (51) = 19.42, *p* < 0.001) and for part two (t (19) = 10.69, *p* < 0.001). We then used a repeated-measure ANOVA to compare the confidence level of participants between different experimental parts (within factor) and to examine if these are dependent on posture order (between factor). There was no significant difference in the confidence levels between parts (F (1,18) = 0.57, *p* = 0.461, ${{\upeta }^{2}}$ < 0.001) and there was no interaction between the experimental part and posture order (F (1,18) = 0.02, *p* = 0.882, ${{\upeta }^{2}}$ < 0.001).

### Mapping of touch after the switch is gravitational or arm-centered

**Figure 4:**
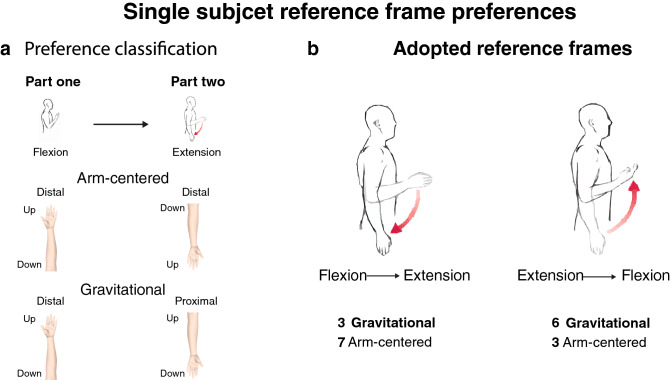
Individual reference frame preferences after switching postures. (**a**) We classified individual preferences as gravitational or arm-centered. For example, a participant in a Flexion posture had a distal coordinate selection in part one, and a proximal selection in an Extension posture in part two. These responses fit a mapping aligned with the gravitational axis (ceiling/head is up). In contrast, a distal selection in part one and a distal selection in part two indicate an arm-centered preference. (**b**) Individual preferences by starting posture. In sum, 10 participants had an arm-centered preference, and 9 participants had a gravitational preference. We corrected all statistical tests for multiple comparisons.

To investigate the reference frame choices before and after switching postures, we analyzed individual participant responses across experimental parts. In each part, we tested whether the number of responses fitting a distal or proximal coordinate was significantly above chance using a t-test. All 52 participants showed a clear and significant preference in part one and 19 out of 20 participants passed the FDR correction with a significant preference in part two. To observe the effect of switching postures, we classified participants’ behavior based on the assumed mapping after the switch (Fig. 4a). We classified the mapping as either gravitational and outside the arm, or as an arm-centered mapping that is anchored to the anatomy of the inner arm. After the switch, 7 participants who started in the Flexion posture adopted an arm-centered mapping and 3 took a gravitational mapping. Of participants whose first posture was Extension, 3 adopted an arm-centered reference frame and 6 a gravitational one (Fig. 4b). We next conducted a post hoc analysis to examine if the 10 participants who adopted an arm-centered reference did so before or after switching postures. A paired t-test showed that the proportion of responses that match a gravitational mapping in part one was significantly higher than part two (t (18) = 3.68, *p* = 0.005, CI [0.27, 1]). This result shows that the change to an arm-centered mapping occurred as a consequence of the posture switch.

## Discussion

The current study investigated the role of proprioception mapping tactile sensations to a visual image by measuring the effects of posture on reference frame selection. With the use of a visual-to-tactile Sensory Substitution Device (SSD), we asked participants to map vibrotactile stimulations delivered on their arms to a visual image and report its spatial location or orientation (towards the upper/lower bounds of the image). Importantly, we asked participants to change arm postures between blocks. Participants’ responses demonstrated their assumed reference frame in the mapping of tactile sensations. We found that participants’ initial reference frame was dependent on their posture, and not anchored to a specific anatomical location on the inner arm, such as the wrist or the elbow, but matches to a gravitational axis (Fig. 2). This mapping also aligns with other body parts, such as the trunk or face that reflect an egocentric reference frame or otherwise to the surrounding environment in an allocentric reference frame^1,4,9,10,17^. Of note, the neutral posture (Fig. 2) could not prompt a similar gravitational reference frame as the arm is perpendicular to the trunk and the up-down coordinates of the room. Still, participants were individually consistent in their responses, even in this ambiguous spatial position.

Participants’ behavior after switching posture took on an interesting pattern. According to a sensorimotor contingencies prediction, switching postures should follow an adaptation period while stored body representations integrate new sensory information^13,16^. The cognitive cost in adapting to new postures is a multisensory integration problem that requires updating stored representations with new information from many modalities. Thus, a sensorimotor account would thus predict inconsistent responses after the switch. In line with this prediction, we indeed find that there is a significant difference in responses that fit the gravitational axis after the switch (Fig. 3). Yet, about half of the group keep the gravitational reference frame while the other half adopt a reference frame that is centered on the anatomy of the inner arm (Fig. 4). Individual changes to reference frame choices could thus explain the reduction in responses that map tactile sensations to the gravitational axis, rather than adaptation to new proprioceptive cues. Most importantly, we observe little cost associated with adopting a new reference frame or maintaining an old one. Participants exhibit a strong consistency in their responses after changing postures, questioning the predictions of the sensorimotor account. We did find that the starting posture might bias the choice of reference frame after the switch, but with no discernable effects on consistency. When considering the weighing scheme model of sensorimotor contingencies^2^ in the context of our findings, the ability to select multiple reference frames with little cognitive costs follows an extreme instance in which all options are of equal weight. Instead, we suggest that participants’ explicit choices of reference frame can supersede incoming proprioceptive information. In part one, participants are not given any information on the vertical axis, and their choices are implicit. Preferences in this initial posture are aligned with a gravitational reference frame and are dependent on posture (Fig. 3). After switching postures, some participants explicitly decide on an arm-centered reference frame by considering the anatomical implications of their early responses, as evident by their verbal justifications (Supplementary table S1). Taken together, our results show that top-down modulation can restrict bottom-up proprioceptive cues when choosing between reference frames and that previously-stored representations could supersede current sensory inputs.

We suggest this behavior is owing to the spatial task’s specific attributes and the stimulated body part. Here, we deliberately chose an anatomical surface without a directional vertical axis (no clear ‘up’ or ‘down’). Also, previous studies made use of complex stimuli such as letters or numbers that include high-level processing and the usage of explicit tasks^3,4,10,14,15,28,29^. In contrast, we used low-level stimuli consisting of horizontal and diagonal lines that require less cognitive demand. Future studies could investigate the extent to which our results change if we deliver stimuli on a different body part with a clear directional vertical axis (e.g., the legs) coupled with a demanding cognitive task. In general, our results could reflect a subtle process whereby proprioception plays a decisive role under implicit conditions (such as arm flexion or extension) but can otherwise be overridden by an explicit choice^4,10,28,29^. Though we did not test for the effects of vision on our task, we propose that the lack of visual inputs facilitated the lack of cognitive costs in switching postures. Vision is essential in forming body representations and has been widely reported as dominant over competing inputs from other modalities^2,22,30–32^. For example, crossing effects in temporal order judgments are decreased when participants are blindfolded^33–35^. Visual cues can thus act both as a promoter for body representations but also as a disturbance to maintaining a stored representation. As our participants wear a blindfold, vision could not override the changes in proprioceptive signals, revealing the distinctive contribution of proprioception.

Proprioception is a unique sensory modality, and though its physiology is well studied, it remains a somewhat esoteric sensory modality. While vision is an exteroceptor identified with a known sensation, proprioception is an interoceptor that, for the most part, is not consciously perceived^27^. In self-initiated body movements, proprioception serves as a perception of the self that results from actions taken and initiated by the self and is thus predictable. As such, we can infer the sensory consequences of arm movement and they interfere less with higher body representations. In conclusion, the present study demonstrates that top-down modulations can offset new proprioceptive information while mapping tactile sensations to a visual image, ultimately showing that proprioception is less influential in the process than previously conceived.

## Supplementary Information

Supplementary Figure and Tables.

## Data Availability

The datasets generated and analyzed during the current study are available in the Open Science Framework repository, https://osf.io/2f4xd.

## References

[1] Serino A, Haggard P (2010). Touch and the body. Neurosci. Biobehav. Rev..

[2] Heed T, Buchholz VN, Engel AK, Roeder B (2015). Tactile remapping: from coordinate transformation to integration in sensorimotor processing. Trends Cogn. Sci..

[3] Prather SC, Sathian K (2002). Mental rotation of tactile stimuli. Cogn. Brain Res..

[4] Parsons LM, Shimojo S (1987). Perceived spatial organization of cutaneous patterns on surfaces of the human body in various positions. J. Exp. Psychol. Hum. Percept. Perform..

[5] Pritchett LM, Carnevale MJ, Harris LR (2012). Reference frames for coding touch location depend on the task. Exp. Brain Res..

[6] Badde S, Heed T, Röder B (2016). Integration of anatomical and external response mappings explains crossing effects in tactile localization: a probabilistic modeling approach. Psychon. Bull. Rev..

[7] Shelton AL, McNamara TP (2001). Systems of spatial reference in human memory. Cogn. Psychol..

[8] Medina J, Coslett HB (2010). From maps to form to space: touch and the body schema. Neuropsychologia.

[9] Harris LR (2015). How our body influences our perception of the world. Front. Psychol..

[10] Hartcher-O’Brien J, Auvray M (2016). Cognition overrides orientation dependence in tactile viewpoint selection. Exp. Brain Res..

[11] Kelly JW, Avraamides MN, Loomis JM (2007). Sensorimotor alignment effects in the learning environment and in novel environments. J. Exp. Psychol. Learn. Mem. Cogn..

[12] Longo MR (2015). Implicit and explicit body representations. Eur. Psychol..

[13] Badde S, Röder B, Heed T (2015). Flexibly weighted integration of tactile reference frames. Neuropsychologia.

[14] Sekiyama K (1991). Importance of head axes in perception of cutaneous patterns drawn on vertical body surfaces. Percept. Psychophys..

[15] Arnold G, Spence C, Auvray M (2016). Taking someone else’s spatial perspective: natural stance or effortful decentring?. Cognition.

[16] Badde S, Heed T (2016). Towards explaining spatial touch perception: weighted integration of multiple location codes. Cogn. Neuropsychol..

[17] Azañon E, Stenner MP, Cardini F, Haggard P (2015). Dynamic tuning of tactile localization to body posture. Curr. Biol..

[18] Arnold G, Auvray M (2014). Perceptual learning: tactile letter recognition transfers across body surfaces. Multisens. Res..

[19] Cadieux ML, Shore DI (2013). Response demands and blindfolding in the crossed-hands deficit: an exploration of reference frame conflict. Multisens. Res..

[20] Lawson R, Boylan A, Edwards L (2014). Where you look can influence haptic object recognition. Atten. Percept. Psychophys..

[21] Harrar V, Harris LR (2010). Touch used to guide action is partially coded in a visual reference frame. Exp. Brain Res..

[22] Longo MR (2014). The effects of immediate vision on implicit hand maps. Exp. Brain Res..

[23] Maidenbaum S (2014). The " EyeCane ", a new electronic travel aid for the blind: technology, behavior & swift learning. Restor. Neurol. Neurosci..

[24] Chebat D-R, Maidenbaum S, Amedi A (2015). Navigation using sensory substitution in real and virtual mazes. PLoS ONE.

[25] Chebat, D.-R., Maidenbaum, S. & Amedi, A. The transfer of non-visual spatial knowledge between real and virtual mazes via sensory substitution. *2017 Int. Conf. Virtual Rehabil.* 1–7 (2017).

[26] Abboud S, Hanassy S, Levy-Tzedek S, Maidenbaum S, Amedi A (2014). EyeMusic: introducing a ‘visual’ colorful experience for the blind using auditory sensory substitution. Restor. Neurol. Neurosci..

[27] Proske U, Gandevia SC (2012). The proprioceptive senses: their roles in signaling body shape, body position and movement, and muscle force. Physiol. Rev..

[28] Volcic R, Wijntjes MWA, Kappers AML (2009). Haptic mental rotation revisited: multiple reference frame dependence. Acta Psychol. (Amst.).

[29] Hartcher-OBrien J, Auvray M (2014). The process of distal attribution illuminated through studies of sensory substitution. Multisensory Research.

[30] Azañón E, Soto-Faraco S (2007). Alleviating the ‘crossed-hands’ deficit by seeing uncrossed rubber hands. Exp. Brain Res..

[31] Blanke O (2012). Multisensory brain mechanisms of bodily self-consciousness. Nat. Rev. Neurosci..

[32] Schinazi VR, Thrash T, Chebat D-R (2015). Spatial navigation by congenitally blind individuals. Wiley Interdiscip. Rev. Cogn. Sci..

[33] Röder B, Rösler F, Spence C (2004). Early vision impairs tactile perception in the blind. Curr. Biol..

[34] Crollen V, Albouy G, Lepore F, Collignon O (2017). How visual experience impacts the internal and external spatial mapping of sensorimotor functions. Sci. Rep..

[35] Heed T, Backhaus J, Röder B, Badde S (2016). Disentangling the external reference frames relevant to tactile localization. PLoS ONE.

