# Supplementary material for: Applying a novel visual-to-touch sensory substitution for studying tactile reference frames
Source: Sci Rep. 2021 May 20;11:10636. doi: 10.1038/s41598-021-90132-7 (PMC8137949; doi:10.1038/s41598-021-90132-7)
Supplement: Supplementary file 1 — Supplementary Figure and Tables. [file 41598_2021_90132_MOESM1_ESM.docx]

Applying a Novel Visual-to-Touch Sensory Substitution for Studying Tactile Reference Frames

Or Yizhar*^1,2^, Galit Buchs^1,2^, Benedetta Heimler^2^, Doron Friedman^3^, Amir Amedi^2^

**^1^**Department of Cognitive Sciences, The Hebrew University of Jerusalem, Israel, **^2^**Baruch Ivcher School of Psychology, Interdisciplinary Center Herzliya, Israel, **^3^**Center of Advanced Technologies in Rehabilitation (CATR), Sheba Medical Center, Ramat Gan, Israel, **^4^**Sammy Ofer School of Communications, Interdisciplinary Center Herzliya, Israel

SUPPLEMENTARY DATA

**
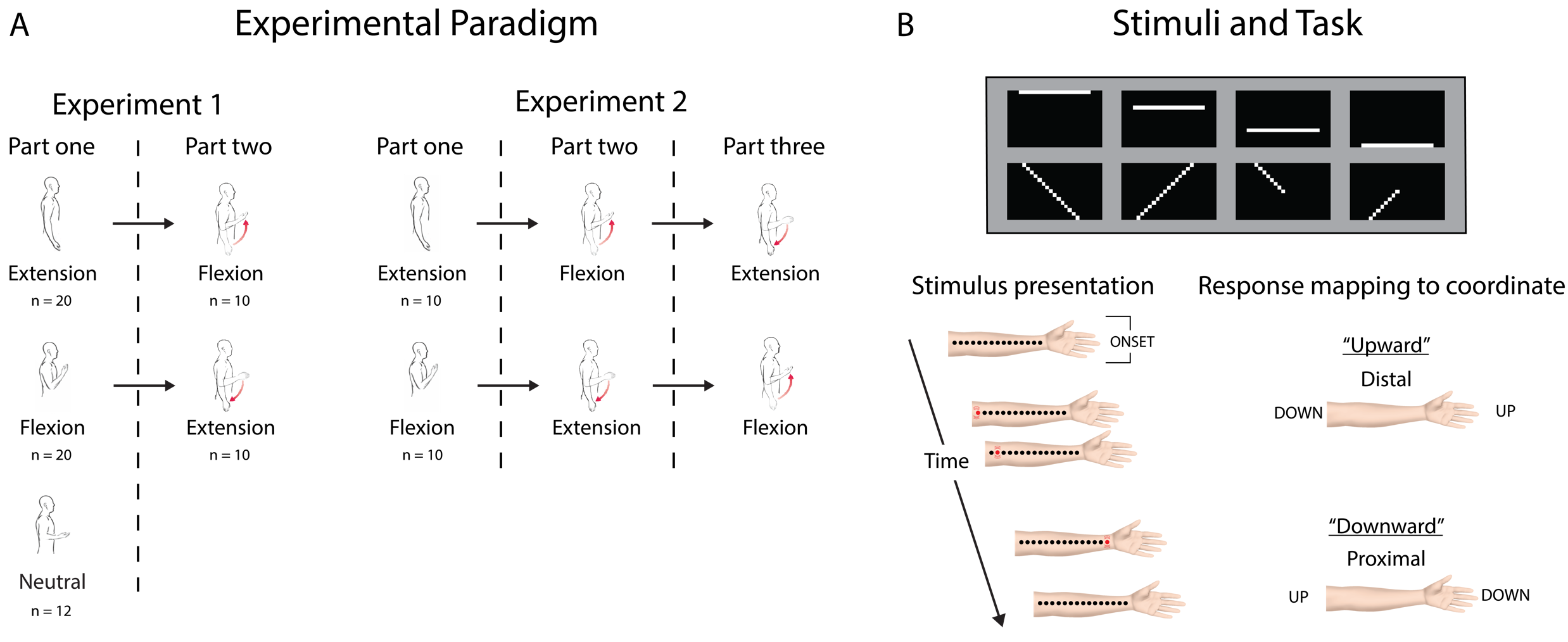
**

**Supplementary Fig. S1.** At the end of the experiment, we presented a few participants with an additional stimulus, before removing their blindfold. The stimulus consisted of a diagonal line as depicted in the stimulus presentation. After removing the blindfold, participants drew the stimulus on a paper, within a closed box representing the image's borders. We used this procedure as a proof of concept that participants indeed transformed the tactile sensations into visual images. Four such examples are attached in the drawings below.

| *Subject from Experiment 1 – Flexion posture*  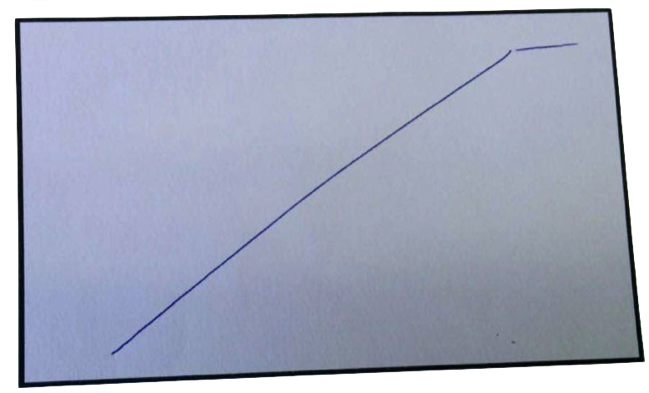 | *Subject from Experiment 1 – Flexion posture*  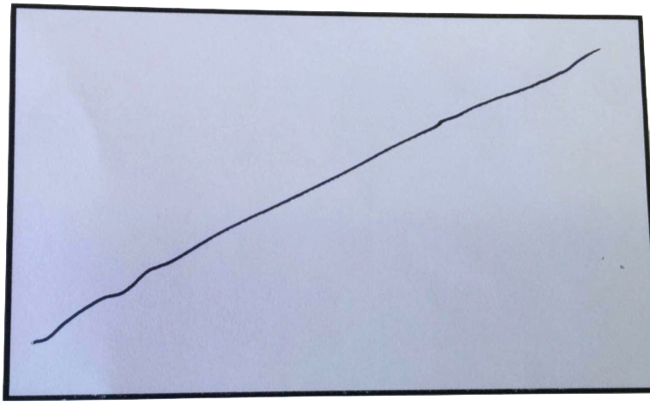 |
| --- | --- |
| *Subject from Experiment 1 – Flexion posture*  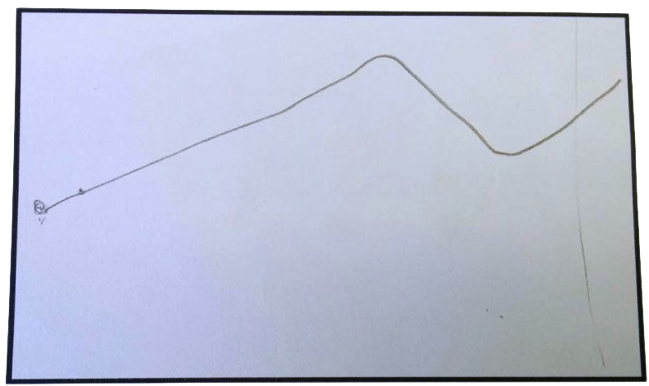 | *Subject from Experiment 1 – Extension posture*  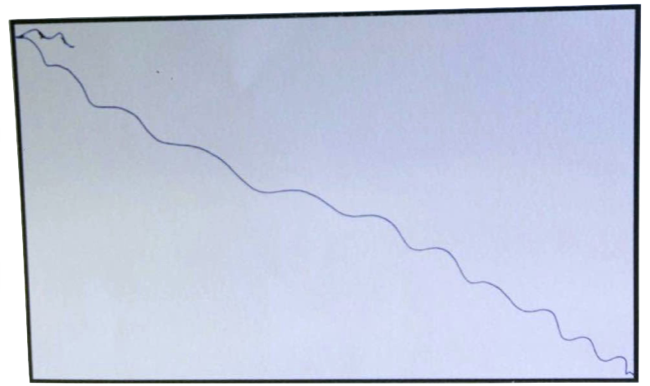 |

SUPPLEMENTARY TABLES

**Supplementary** **Table S1.** After completing the experiment, we asked participants *“how did you decide what is up and what is down in the image?”*. We collected their verbal responses (Hebrew) in writing. Presented here are 16 such examples from participants who completed both experimental parts, and a further 6 responses from the Neutral posture in part one.

| **Condition** | **Subject** | **Verbal Response** |
| --- | --- | --- |
| Flexion  ↓  Extension | S1 | *Palm was up, after switching decided to be consistent with my former choice, it took her a few seconds to adapt* |
|  | S2 | *Decided to stick to her first preference after switching posture (“wrist is up”). Got a little confused after the switch but quickly adapted* |
|  | S3 | *Drew an imaginary line with the arm, after switching posture consciously decided that up is towards the head and ceiling* |
|  | S4 | *Decided that the floor was down, every time he changed posture he mapped his responses accordingly* |
|  | S5 | *Decided first according to the relation between the arm and the floor, afterwards decided to be consistent in his answers even though he felt the directions have flipped* |
|  | S6 | *Initially went by her “gut feeling”, then decided to stick to her former choice after the switch* |
|  | S7 | *Decided based on the relation between the arm’s position and the floor* |
|  | S8 | *Initially decided that palm is up and chose to stick with it after the switch* |
| Extension  ↓  Flexion | S9 | *First decided "naturally" that elbow is up. Wanted to be consistent and decided to keep elbow as up after she switched postures.* |
|  | S10 | *First decided that "up" will align to the whole body and so wrist was "down", decided to be consistent with the latter after switching postures* |
|  | S11 | *Decided that up is what is closer to the elbow because that is "up" for the whole body, wanted to be consistent after changing postures* |
|  | S12 | *First decided to that up should be the same is in the room, after switching postures decided to be consistent in her answer* |
|  | S13 | *Imagined the task as drawing on a piece of paper, and so what is up in space corresponds to up in the drawing* |
|  | S14 | *Considered how “high” the stimuli were* |
|  | S15 | *Decided to keep the same coordinate as first posture as it seemed easier* |
|  | S16 | *Decided according to the relation between the earth and the arm* |
| Neutral (part one) | S17 | *Up was towards the hand because there was the first vibration* |
|  | S18 | *Away from the body was down and as the vibration is closer to the torso it felt like up* |
|  | S19 | *Imagined the arm as if it was page and the bottom part was the wrist* |
|  | S20 | *As if I was looking at a graph that is directed towards the finger* |
|  | S21 | *Imagined the arm as pointing upwards and so the elbow was down* |
|  | S22 | *Decided randomly that up is towards the wrist, considered switching while in posture but decided to be consistent* |

**Supplementary** **Table S2.** One-way ANOVA between poste (proportion distal responses)

|  | *SS* | *df* | *F* | *P-value* | *Eta squared* |
| --- | --- | --- | --- | --- | --- |
| Posture | 7.191 | 2 | 48.937 | <0.001 | 0.666 |
| Error | 3.6 | 49 | 1.000 | 0.500 | 0.334 |

Postures – Extension, Flexion, Neutral

**Supplementary** **Table S3.** Repeated measures ANOVA between first and second posture (proportion gravitational preference)

|  | *SS* | *df* | *F* | *P-value* | *Eta squared* |
| --- | --- | --- | --- | --- | --- |
| Blocks | 1.477 | 1 | 13.98 | 0.002 | 0.353 |
| Interaction | 0.809 | 1 | 7.648 | 0.013 | 0.193 |
| Error | 1.903 | 18 | 1.000 | 0.500 | 0.454 |

Posture order - (Flexion-extension / Extension – flexion), Blocks - first posture, second posture

**Supplementary** **Table S4.** Repeated measures ANOVA between first and second posture (consistency index)

|  | *SS* | *df* | *F* | *P-value* | *Eta squared* |
| --- | --- | --- | --- | --- | --- |
| Blocks | 0.002 | 1 | 0.567 | 0.461 | <0.001 |
| Interaction | <0.001 | 1 | 0.023 | 0.882 | <0.001 |
| Error | 0.078 | 18 | 1.000 | 0.500 | 0.018 |

Posture order - (Flexion-extension / Extension – flexion), Blocks - first posture, second posture

**Supplementary** **Table S5.** Group consistency levels calculated for each block

|  | *n* | *Mean* | *SD* | *P-value* |
| --- | --- | --- | --- | --- |
| First posture | 52 | 0.45 | 0.074 | <0.001 |
| Second posture | 20 | 0.441 | 0.08 | <0.001 |
